# Dynamic Network Driver Analysis Identifies Master Factors Associated with Progression of Solar Lentigines

**DOI:** 10.3390/biology14070876

**Published:** 2025-07-17

**Authors:** Deyu Cai, Hong Zhang, Chengming Zhang, Xue Xiao, Xiao Cui, Xuelan Gu, Luonan Chen

**Affiliations:** 1 School of Life Science and Technology, ShanghaiTech University, Shanghai 201210, China; caidy@shanghaitech.edu.cn; 2 Key Laboratory of Systems Biology, Shanghai Institute of Biochemistry and Cell Biology, Center for Excellence in Molecular Cell Science, Chinese Academy of Sciences, Shanghai 200031, China; 3 Unilever Research & Development Centre Shanghai, Shanghai 200335, China; hong-sh.zhang@unilever.com (H.Z.); xue.xiao@unilever.com (X.X.); sammy.cui@unilever.com (X.C.); 4 International Research Center for Neurointelligence, The University of Tokyo Institutes for Advanced Study, The University of Tokyo, Tokyo 113-0033, Japan; zhangchengming@g.ecc.u-tokyo.ac.jp; 5 Key Laboratory of Systems Health Science of Zhejiang Province, School of Life Science, Hangzhou Institute for Advanced Study, University of Chinese Academy of Sciences, Chinese Academy of Sciences, Hangzhou 310024, China; 6 School of Mathematical Sciences, Shanghai Jiao Tong University, Shanghai 200240, China; 7 School of Artificial Intelligence, Shanghai Jiao Tong University, Shanghai 200240, China

**Keywords:** solar lentigines, Dynamic Network Driver, network control, skin pigmentation

## Abstract

Solar lentigines, commonly caused by prolonged ultraviolet exposure, raise the risk of skin disorders and remain challenging to manage due to their complex mechanisms. Understanding the molecular mechanisms driving the progression of solar lentigines is crucial for developing effective protective strategies. In this study, we introduced a novel method, Dynamic Network Driver (DND), which identifies upstream regulators that drive disease progression by integrating the Dynamic Network Biomarker (DNB) approach with network control theory. By applying DND to multi-omics data from solar lentigines subjects, we (1) identified the key drivers associated with solar lentigo progression, with their functions involved in differentiation and dermal–epidermal junction; and (2) highlighted *ARNT2* and *TBX2* as significant master factors supported by in vitro validation in melanocytes and pigmented 3D living skin equivalent models. These results demonstrate the potency of DND for uncovering the molecular mechanisms behind solar lentigines and informing therapeutic strategies. In summary, the DND approach identified novel drivers of solar lentigo progression, acting as new markers for spot mitigation in 3D spot mimic models.

## Simple Summary

Solar lentigines (also known as age spots) are common skin lesions caused by prolonged sun exposure. They are characterized by excessive pigmentation and structural changes at the skin’s dermal–epidermal junction. Although many molecular factors have been associated with the formation of these spots, the mechanisms driving their progression remain unclear. In this study, we developed a novel systems biology approach called Dynamic Network Driver (DND), which combines network control theory with time-dependent network fluctuation analysis. By applying DND to mRNA and microRNA data from human skin samples at different stages of solar lentigines, we identified the key regulatory genes involved in spot progression. Among them, ARNT2 and TBX2 were highlighted and validated using melanocyte cultures and 3D skin models. Our findings offer new insights into the dynamic regulation of solar lentigines and suggest potential targets for future preventive or therapeutic strategies.

## 1. Introduction

Skin hyperpigmentation spots result from excessive melanin production, often triggered by UV exposure [1]. Among them, solar lentigines (age spots) are small, well-circumscribed patches characterized by hyperpigmentation, elongated rete ridges with melanocytes, and dermal–epidermal junction perturbations [2]. Electron microscopy reveals larger melanosome complexes in keratinocytes [3]. Cario-Andre et al. classified solar lentigines into three histopathological stages, with the most severe stage showing deep rete ridges and bud-like epidermal growths, reflecting progressive pigment accumulation, melanocyte activation, and the elongation of rete ridges [4].

The mechanisms underlying solar lentigines have been explored through in vitro and in vivo experiments and conventional bioinformatics analysis such as Genome-Wide Association Studies (GWASs) and transcriptomics analysis. Key factors such as KGF, HGF, SCF, and EDN1 (ET-1) were reported to contribute to the solar lentigo development [5,6,7,8]. GWASs identified genetic variants linked to pigmented spots, including *IRF4*, *MC1R*, and *BNC2*, while studies in Korean females highlighted *CDKN2B-AS1* and *MFSD12* [9,10]. Transcriptomic analyses revealed significantly changed genes and their involvement in melanogenesis, melanocyte activation, dermal–epidermal junction disruption, and keratinization, further supporting the role of these molecular pathways. Despite these insights, the mechanisms driving spot progression remain largely unstudied.

Unlike conventional differential expression or association analysis, Dynamic Network Biomarker (DNB) analysis integrates temporal expression and network information to identify biomarkers associated with disease progression [11]. An improved approach, landscape DNB (l-DNB), enhances this approach by incorporating single-sample network (SSN) analysis, enabling robust predictions with limited samples [12]. Zhang et al. applied l-DNB to time-series transcriptome data from a 3D skin model, revealing key DNB genes like *COL7A1* and *CTNNB1* in UVB-induced skin lightening [13]. Additionally, network-based methods such as the minimum Feedback Vertex Set (mFVS) have been employed to uncover disease biomarkers and guide network control strategies, demonstrating superior performance in cancer gene detection [14].

**Figure 1:**
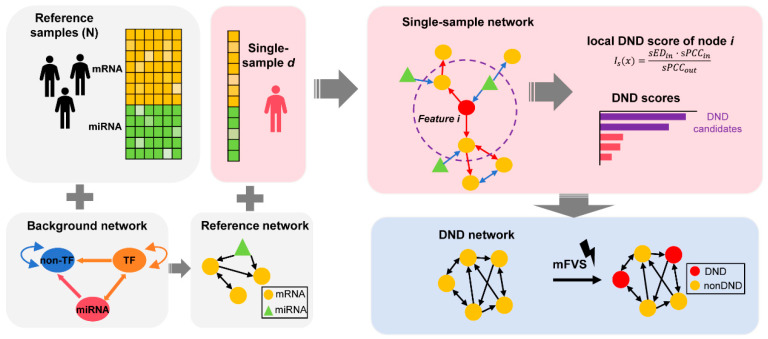
Schematic representation of the Dynamic Network Driver (DND) framework. The DND framework begins by constructing a reference network using control (non-lesional) samples, integrated with a biologically curated background regulatory network that incorporates known molecular interactions. For each individual (lesional) sample, a single sample network (SSN) is built, which includes directional information derived from prior knowledge. The SSN is constructed using statistical perturbation analysis, which quantifies sample-specific deviations from the reference group. This allows us to accurately capture individual-specific molecular network changes, forming the theoretical foundation of the method. Next, DND scores are computed for each node (gene or regulator) to reflect their influence on network dynamics. Top-ranking features are selected to form the DND network, which highlights the putative regulators driving disease progression. Finally, the minimum Feedback Vertex Set (mFVS) algorithm is applied to the DND network to identify a minimal set of key nodes (DNDs) whose removal would disrupt all feedback loops in the system. These core DNDs represent potential upstream drivers in the regulatory architecture of solar lentigines.

To comprehensively understand the progression of solar lentigines, it is essential to integrate temporal dynamics and molecular networks to identify key drivers. Here, multi-omics data (mRNA and microRNA) from solar lentigines were analyzed using systems biology approaches to explore the underlying mechanisms and propose key master regulators for spot progression. In particular, Dynamic Network Driver (DND) is proposed here, which integrates directed network analysis into l-DNB by incorporating both downstream node-based DNB scoring and mFVS-based key module prioritization. The combination of these two components allows DND to identify the critical regulatory factors driving spot progression (Figure 1). Through DND analysis, we identified *ARNT2* and *TBX2* as potential drivers of solar lentigo progression and validated their roles through in vitro experiments. This comprehensive approach provides a deeper understanding of the molecular mechanisms driving solar lentigines, facilitating the development of in vitro models distinct from general pigmentation studies and informing preventive strategies.

## 2. Materials and Methods

### 2.1. Sample Collection and Data Generation

This study used the same dataset as the Rocha et al. study [15]. This study was approved by the Allendale Investigational Review Board. All subjects provided informed consent. Twenty Caucasian females aged 45–65 with spots, as diagnosed by a Board-Certified Dermatologist (TKL Research, Inc., Fair Lawn, NJ, USA), were enrolled. Biopsy samples were collected from photo-protected sites, spot sites (lesion), and peri-lesional sites. Peri-lesional samples were collected from an area close to the spot. All samples were immediately stored in RNAlater solution (Ambion, Austin, TX, USA). An mRNA microarray was conducted by Illumina’s HumanHT-12 Expression BeadChip (Illumina, San Diego, CA, USA). An miRNA array was conducted by Exiqon Inc. (Vedbaek, Denmark).

Dermatoscopic images were collected prior to biopsy sampling using SIAscope V (Astron Clinica, Cambridge, UK). These images were processed to evaluate the spot size and melanin index.

### 2.2. Data Pre-Processing and Directed Background Network Construction

A total of 168 skin samples were initially collected from 20 subjects. A total of 156 high-quality samples passed stringent quality control. To ensure data quality and sufficient statistical power, we applied a two-step outlier detection procedure to the combined mRNA and miRNA expression matrix. First, principal component analysis (PCA) was performed and 95% confidence ellipses were drawn for each disease stage; samples falling outside these ellipses (subjects 11, 12, and 14) were flagged as potential outliers (Supplementary Figure S1a). Second, we calculated Mahalanobis distances in the same PCA space—thereby accounting for the covariance structure of the data—and flagged any sample exceeding the 95% confidence threshold (subjects 1, 3, 7, 11, 12, 14, and 17), as shown in Supplementary Figure S1b. To maximize stringency, we removed all samples from subjects 11, 12, and 14, yielding a final dataset of 136 samples for downstream analysis. We then evaluated statistical power via PERMANOVA on Euclidean distance matrices of gene expression profiles before and after outlier removal. Group differences among the three stages remained highly significant (original: F = 2.41, R^2^ = 3.06%, *p* = 0.001; filtered: F = 3.04, R^2^ = 4.38%, *p* = 0.001), indicating that sample exclusion enhanced the explained variance without compromising statistical inference. To reduce the dimensionality of the features, we applied a three-part filtering strategy: (1) we selected features related to phenotype data via Pearson correlation coefficient (PCC); (2) we selected features associated with the skin from relevant databases: DIANA-miTED and The Human Protein Atlas [16,17]; and (3) we selected features associated with symptom stages using ANOVA. In total, 5273 features passed through this filtering process and were used for further analysis.

The background network was constructed by integrating multiple data sources that provide directional and causal information at varying confidence levels. The directed protein–protein interaction network (dPPI) [18] was derived from yeast two-hybrid data and curated databases, with directionality inferred via a Bayesian learning framework. Context-specific gene regulatory networks from the GRAND database [19] were incorporated, inferred by integrating prior knowledge and gene expression profiles—using established network inference algorithms such as PANDA [20] and LIONESS [21]. Additionally, experimentally validated miRNA–target interactions were included from miRTarBase [22]. This integration of both computationally inferred and experimentally supported interactions provides a comprehensive, directed regulatory network for downstream analysis.

### 2.3. Dynamic Network Driver (DND) Analysis

The DND method consists of two parts: directed l-DNB and network control. Initially, we concatenate the normalized mRNA and miRNA expression data into a unified matrix, which is then processed using the directed l-DNB method. Results were filtered by mFVS to obtain the final output. The former mainly consists of two parts: single-sample network construction and DND score calculation.

### 2.4. Single-Sample Network (SSN) Construction

The reference network was constructed firstly by mapping reference samples to the directed background network using the integrated expression matrix of normalized mRNA and miRNA expression data. The edge of the reference network was predicted by calculating the ${PCC}_{n}$ for feature pairs from control samples, and the *p*-value of PCC was used to identify significantly correlated feature pairs.

$$PCC_{n}(x,y)=\frac{\sum_{i=1}^{n}\left(x_{i}-\bar{x})(y_{i}-\bar{y}\right)}{\sqrt{\sum_{i=1}^{n}(x_{i}-\bar{x}{)}^{2}}\sqrt{\sum_{i=1}^{n}(y_{i}-\bar{y}{)}^{2}}}$$

where $x_{i}$ and $y_{i}$ are the expression levels of features x and y in the reference sample i, respectively, and $\bar{x}$ and $\bar{y}$ are the average expression levels for features x and y in reference samples with a sample size of n.

The SSN approach was adopted to construct the network of samples from individual subjects [23]. The SSN of a single sample was constructed by the difference of the network constructed using reference data and the network using reference data combined with the new single sample.

$$sPCC(x,y)={PCC}_{n+1}(x,y)-{PCC}_{n}(x,y).$$

The statistical hypothesis test (Z-test or U-test) was applied to test whether feature x and feature y were significantly correlated at the single-sample level.

### 2.5. DND Score Calculation

The DND score was calculated by applying the original DNB score calculation to the directed network. Here, the DND score of each feature was calculated first. During generation, the local DNB score $I_{s}$ for node x within the network (mRNA or miRNA feature) can be derived via

$$I_{s}(x)=\frac{sED_{in}\cdot sPCC_{in}}{sPCC_{out}}$$

Here, $sED_{in}$ represents the average deviation in the expression of all of the $\left(1+N_{x_{d}}\right)$ nodes in the local module of node x for sample d relative to the control samples. The $sPCC_{in}$ for the local module of node x is the average value of the single-sample PCC of node x and its first-order neighbors $N_{x_{d}}$. $sPCC_{out}$ is defined as the average correlation between the inner and outer nodes of the local module for node x.

The DNB satisfies the following three conditions: first, there is significant deviation fluctuation among the first-order neighbors in the SSN; second, the correlation between node x and its first-order neighbors significantly increases; and third, the correlation between first-order and second-order neighbors significantly decreases.

The DND score is calculated by only incorporating the downstream node of node x when calculating the $sPCC_{in}$ and $sPCC_{out}$. Therefore, the DND score evaluates the driving effect of each node at the directed network. Here the DND score of each feature, mRNA and miRNA, was calculated and combined, so the DND was the score for the individual sample.

### 2.6. DND Prioritization Using Network Control Analysis

The Minimal Feature Vector Selection (mFVS) method is a network control analysis approach that helps identify key regulatory genes and the minimal modules that contribute to network stability. mFVS is particularly valuable for understanding the controllability and stability of complex biological networks, where feedback loops often govern critical system behaviors. By identifying the minimal set of features that control the network’s behavior, mFVS enables researchers to focus on the most impactful genes in a biological system.

In our study, mFVS was applied to the subnetwork of DND candidates in IPA to pinpoint the smallest module within this network. This method allowed us to prioritize key driver features and narrow down the list of potential drivers involved in the progression of solar lentigines.

### 2.7. Biological Function Analysis

Pathway and upstream factor analysis were conducted using Ingenuity Pathway Analysis (IPA, QIGEN, Redwood City, CA, USA). GO enrichment analysis was conducted using R (version 4.4.2) package clusterProfiler (version 4.14.6) [24].

### 2.8. Application of Skin Models for Validation

Normal human epidermis melanocytes were supplied by Guangdong Biocell Biotechnology (NHEMs, Lot: MC24112101, Xi’an, China). A reconstructed pigmented 3D living skin equivalent model was provided by Guangdong Biocell Biotechnology (Melakutis^®^, Lot: MS250101, Xi’an, China). Synthetic Endothelin 1 (labeled as ET-1 in the following text; SIGMA-ALDRICH, St. Louis, MO, USA) was used to assess the effect of ET-1. LCC was purchased from Sinerga (HairApp, LCC purity ≥ 98.00%, Varese, Italy).

In the ET-1-treated melanocyte study, the cells were treated with or without 5 nM ET-1 for three days, with the cell culture medium refreshed daily. After treatment, the melanocytes were collected for qPCR analysis.

Melanocyte samples were lysed by AG RNAex Pro Reagent (Accurate Biotechnology, AG21102, Changsha, China) and the total RNA was extracted with chloroform. The extracted RNA was reverse-transcribed to generate the template cDNA with Evo M-MLV RT Premix for qPCR (Accurate Biotechnology, Cat: AG11728, Changsha, China). The expression levels of *ARNT2* (F: 5′-*AAGTAGCGGGCAGTTCCAAG*-3′; R: 5′-*GGTTGGATCTCCTGGCATGG*-3′) and *TBX2* (F: 5′-*TCCTGAAGCTGCCTTACAGC*-3′; R: 5′-*TTGGCAAACGGGTTGTTGTC*-3′) were analyzed using the SYBR^®^ Green Premix Pro Taq HS qPCR Kit (Accurate Biotechnology, AG11701, Changsha, China). Actin beta (*ACTB*) gene was considered as a housekeeping gene, and the expression levels of the target genes were normalized to *ACTB*. The fold changes in gene expression were compared to the NT group.

In the UV- and ET-1-co-stimulated study, Melakutis^®^ (Guangdong Biocell Biotechnology, Xi’an, China) in the ET-1 and UVB co-treatment group was treated with 5 nM ET-1 in the daily refreshed culture medium and the model was exposed to 50 mJ/cm^2^ for 6 days. In the LCC treatment group, 0.2% LCC was applied topically on Day 2, Day 4, and Day 6 before UVB exposure, and the UVB and ET-1 treatment method was kept the same as in the ET-1 and UVB co-treatment group. The non-treatment (NT) group was treated with refreshed culture medium every day. On Day 7, the models were harvested for real-time qPCR analysis. The qPCR procedure remained the same as that described previously. Only the primers of *TYR* (F: 5′-*GGTACAGGGATCTGCCAACG*-3′; R: 5′-*CCCGGTTATGTCCAATGGGT*-3′) and *HMGB1* (F: 5′-*CGGACAAGGCCCGTTATGAA*-3′; R: 5′-*GAGGAAGAAGGCCGAAGGAG*-3′) were added to the test. The melanin distribution was assessed with the Masson–Fontana melanin staining kit (Yike Biotechnology Service Co., Ltd., Cat: YK2318, Xi’an, China) following the same protocol as in a previous study [25].

## 3. Results

### 3.1. Differentiation of mRNA and miRNA Expression Profiles in Solar Lentigines Compared to Photo-Protected Skin

**Figure 2:**
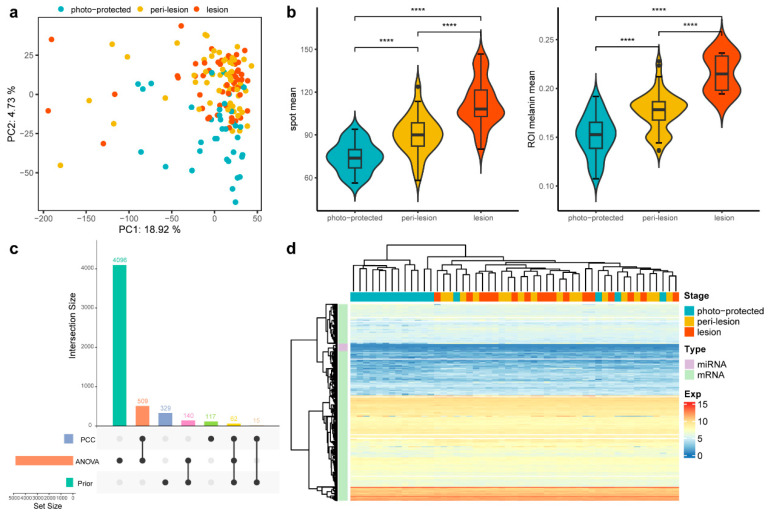
Comparison of expression and phenotypic data across three stages. (**a**) PCA plot of 156 samples across the three stages. Samples from the lesion and peri-lesion stages cluster more closely, while those from the photo-protected stage are more distinct. (**b**) Violin plots of two phenotypic measurements across the three stages. The statistical significance of differences between the groups was determined by the *t*-test (**** *p* ≤ 0.0001). (**c**) UpSet plot to illustrate the composition and intersections of three feature sets used for downstream analysis. The left bar plot represents the number of features in each set, while the top bar plot shows intersection sizes. Black dots and connecting lines indicate the contributing sets for each intersection. (**d**) Heatmap of the expression pattern of the selected features. Subjects at the peri-lesion and lesion stages clustered together, whereas those at the photo-protected stage remained separate.

To investigate the molecular differences underlying solar lentigines, mRNA and miRNA expression across lesion, peri-lesion, and photo-protected skin sites were collected for comparison. Outliers identified through a principal component analysis (PCA) of normalized mRNA and miRNA expression data were excluded from subsequent analyses to ensure robust findings (Figure 2a). Finally, 136 samples were retained for further analysis.

Additionally, phenotypic measurements from 11 subjects were integrated with the omics dataset to enhance biological interpretation. Phenotypic assessments revealed that spot lesion sites exhibited significantly larger pigmented areas and higher melanin content compared to peri-lesion and photo-protected sites, as shown by SIAscope imaging analysis (Figure 2b). After feature selection (Methods), 5273 prioritized spot-associated mRNA and miRNA features were identified, with most of them derived from the ANOVA set rather than from previously well-known features (Figure 2c). mRNA and miRNA expression profiles clearly distinguished solar lentigines (lesion and peri-lesion) from photo-protected skin. However, the expression profiles between lesion and peri-lesion subjects were highly similar, indicating a shared molecular landscape between these regions (Figure 2d). This inspired the conclusion that transcriptomic alterations precede visible phenotypic change and highlighted the importance of DNB theory for early signaling identification.

### 3.2. Identification of DND Candidates for Solar Lentigo Progression

**Figure 3:**
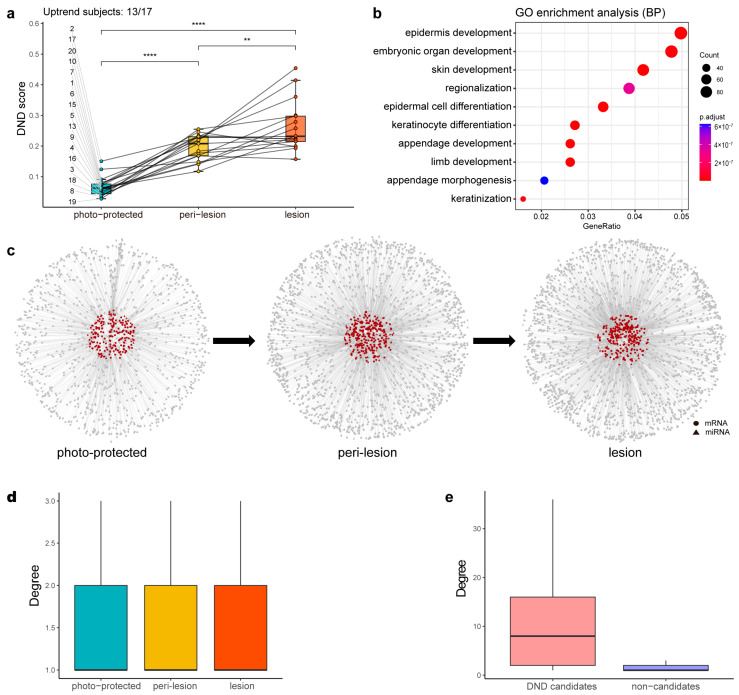
Intermediate results of DND analysis. (**a**) Boxplot showing the distribution of DND scores across the photo-protected, peri-lesion, and lesion stages. Each dot represents the average DND score for a given individual at a given stage, and lines connect samples from the same individual. Among the 17 individuals with complete data, 13 exhibited a monotonic increase across the stages (“Uptrend subjects: 13/17”; subjects 2, 4, 5, 8, 9, 10, 13, 15, 16, 17, 18, 19, and 20). Statistical significance between the stages was assessed using paired *t*-tests. *p* < 0.01 (**), *p* < 0.0001 (****). (**b**) Dot plot of the Gene Ontology Biological Process (GO-BP) enrichment analysis, where the x-axis represents the gene ratio and the y-axis lists the enriched terms. The dot size corresponds to the number of genes associated with each term, and the color represents the enrichment significance (shown as −log10(adjusted *p*-value)). (**c**) Local DND network within the overall SSN for each stage. Circle: mRNA. Triangle: miRNA. Red: DND candidates. Gray: others. (**d**) Boxplot of the degree distribution (i.e., the number of direct connections a node has) of SSNs across the three stages, with no significant difference observed. (**e**) Boxplot of the degree of DND candidates and non-candidates within the lesion-stage SSN, revealing a significant difference that emphasizes the importance of DNDs.

The differences in average DND scores across the three stages (photo-protected, peri-lesion, and lesion) highlight the effectiveness of the DND approach in capturing dynamic regulatory changes during solar lentigo progression (Figure 3a). Each point in the figure represents the mean DND score for an individual at a given stage, with lines connecting measurements within the same subject, demonstrating consistent intra-individual monotonic increases for most subjects (13 out of 17). This within-subject consistency was further supported by paired *t*-tests showing significant stage-wise differences. Moreover, the adjusted intraclass correlation coefficient (ICC) of 0.028 indicates that the vast majority of the variation in DND scores arises from changes across stages within individuals, rather than baseline differences between individuals. These results collectively demonstrate the robustness and reproducibility of the DND score in reflecting biologically meaningful stage-dependent changes rather than inter-individual variability. Based on the average DND scores across the three stages, 387 features, including 70 miRNAs, were defined as DND candidates. Some of these candidates were directly associated with skin function, such as *EGR3*, *KRT10*, and *PPARG*, which contribute to the skin barrier [26,27,28,29], and *COL4A1*, which plays a role in the dermal–epidermal junction [30]. Others were more relevant to molecular mechanisms, such as cell cycle and cell migration. Several miRNAs previously reported to be linked to skin function were also identified as DND candidates, including miRNA-17, miRNA-25, miRNA-203, miRNA-141, miRNA-146, and miRNA-22, as well as miRNAs altered during photoaging, such as miRNA-34 and miRNA-145 [31].

Gene Ontology (GO) enrichment analysis revealed that DND candidates (Supplementary Table S1) were mainly enriched in developmental and differentiation processes, such as epidermis development (GO:0008544) and epidermal cell differentiation (GO:0009913) (Figure 3b). Concurrently, Ingenuity Pathway Analysis (IPA) revealed the significant involvement of DND candidates in critical signaling pathways like Wnt/β-catenin signaling, retinoic acid receptor (RAR), hepatocyte growth factor (HGF), and ERK/MAPK signaling pathways.

The overall SSN of each stage provided a comprehensive view of regulatory relationships, while the local DND networks within the SSNs showed a progressive increase in network density as the condition advanced (Figure 3c). This trend was consistent with increasing DND scores, suggesting a stronger regulatory network structure at later stages. Interestingly, degree distribution analysis across the three networks showed no significant differences (Figure 3d). However, a comparative analysis between DND candidates and non-candidate features demonstrated that DND candidates exhibited significantly higher network connectivity (degree), underscoring their regulatory importance and central role in solar lentigo progression (Figure 3e).

### 3.3. Prioritization of Key Drivers of Solar Lentigo Progression

To narrow down the potential drivers of solar lentigo progression, we conducted an mFVS analysis on the DND candidates within the network framework identified by IPA. This analysis reduced the list of DND candidates to 45 features (DNDs) (Supplementary Table S2), providing a more focused set of potential molecular drivers for further investigation.

**Figure 4:**
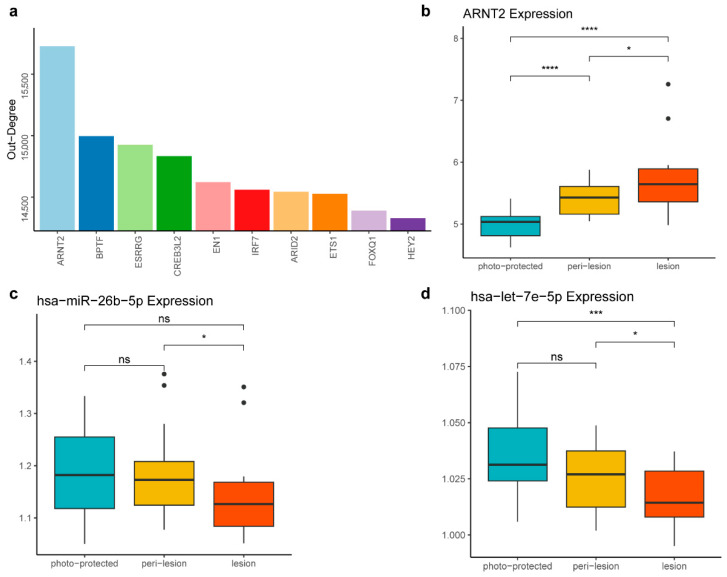
*ARNT2* identified as a candidate therapeutic target for solar lentigines. (**a**) The top 10 DNDs ranked by out-degree in the background network. A higher out-degree in the background network indicates that a feature has more target genes, suggesting it is more likely to be a driver. (**b**) Boxplot showing that *ARNT2* expression levels increase across the three stages. (**c**) Boxplot displaying the decreased expression level of *hsa-miR-26b-5p* across the three stages. (**d**) Boxplot depicting the decreased expression level of *hsa-let-7e-5p* across the three stages. The statistical significance of differences between the groups in panels (**b**–**d**) was determined by the *t*-test (ns, *p* > 0.05; * *p* ≤ 0.05; *** *p* ≤ 0.001; **** *p* ≤ 0.0001).

Among the DNDs, *ARNT2* emerged as the most prominent, ranking highest in out-degree within the background network, underscoring its central role in the regulatory network (Figure 4a). *ARNT2* is a key transcription factor involved in various cellular processes, including responses to oxidative stress, which is crucial in skin aging and pigmentation [32]. Notably, *ARNT2* expression exhibited a consistent upward trend across the three stages of the solar lentigines, indicating its potential involvement in the disease progression (Figure 4b). Furthermore, *TBX2*, another DND, also plays a significant role in cellular differentiation and development. As a member of the T-box transcription factor family, *TBX2* regulates cell proliferation and differentiation-critical processes for tissue homeostasis and repair [33]. The overexpression of *TBX2* has been shown to maintain proliferation and suppress senescence in melanomas [34].

Further investigation into the regulatory dynamics revealed that *ARNT2* is regulated by two miRNAs, *hsa-miR-26b-5p* and *hsa-let-7e-5p*, whose regulatory relationships with ARNT2 are documented in miRTarBase, an informative resource for experimentally validated miRNA–target interactions [22]. Both miRNAs showed a decrease in expression as the stage progressed (Figure 4c,d). The differential expression of these miRNAs suggests they play key roles in modulating *ARNT2* activity, thereby influencing the progression of solar lentigines.

### 3.4. Validation of Gene Expression in Spot Mimic Model

To investigate the association of *ARNT2* and *TBX2* with solar lentigines, normal human epidermal melanocytes and pigmented 3D living skin equivalent models were employed.

**Figure 5:**
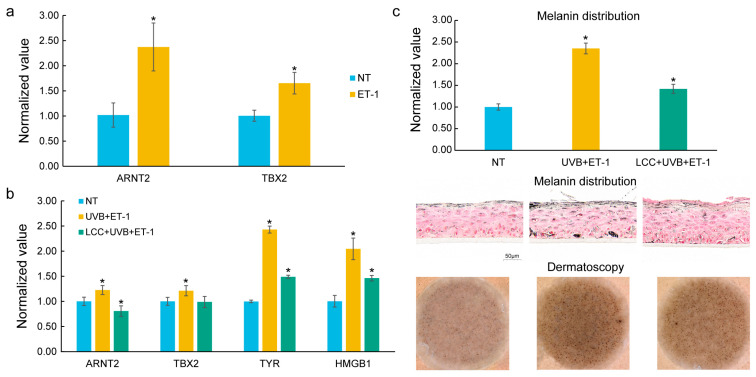
In vitro validation of key genes in melanocytes and a 3D skin model. (**a**) Expression of prioritized genes, *ARNT2* and *TBX2*, in ET-1-treated melanocytes. ET-1 significantly upregulated the expression of *ARNT2* and *TBX2*. (**b**) Expression of key genes in pigmented 3D living skin equivalent models treated with ET-1 and UVB. ET-1 and UVB co-treatment significantly induced the expression of *ARNT2*, *TBX2*, *TYR*, and *HMGB1*. LCC mitigated the induced expression of these genes. (**c**) Melanin distribution and Dermatoscopy images in ET-1/UVB co-treatment models. ET-1 and UVB co-treatment induced hyperpigmentation which was mitigated by LCC. The statistical significance of differences between groups was determined by the *t*-test (* *p* ≤ 0.05); the symbol above the second bar represents the comparison between the first and second bars, while the symbol above the third bar represents the comparison between the second and third bars.

In the melanocyte, ET-1, a growth factor relevant to solar lentigines [7], was used as a stimulator. As compared to NT, ET-1 treatment significantly increased the expression of *ARNT2* and *TBX2* (Figure 5a), which are identified as DNDs.

The pigmented 3D living skin equivalent model was exposed to a UVB and ET-1 co-treatment to mimic the conditions leading to spot formation. Consistent with results from the melanocyte, ET-1 and UVB co-treatment upregulated the expression levels of *ARNT2* and *TBX2*. In addition, ET-1 and UVB co-treatment also notably increased the expression of *TYR*, a critical regulator in melanogenesis [35], and *HMGB1*, an enhancer of melanocyte dendricity [36] (Figure 5b). Furthermore, melanin distribution and Dermatoscopy images confirmed the presence of hyperpigmentation under ET-1 and UVB stimulation (Figure 5c), further corroborating the molecular findings. In the ET-1 and UVB co-treatment models, a skin care technology, Lysine Carboxymethyl Cysteinate (LCC) [37], was applied to assess how it could influence these key genes. Remarkably, LCC treatment effectively mitigated the expression of *ARNT2*, *TBX2*, *TYR*, and *HMGB1*. Additionally, LCC treatment led to a significant reduction in melanin distribution, as shown in histological images and Dermatoscopy images (Figure 5c).

## 4. Discussion

Solar lentigines, commonly induced by UV exposure, are prevalent skin lesions resulting from excessive melanin production. While numerous studies have explored morphological changes and identified marker genes associated with solar lentigines, the molecular drivers of spot progression remain less understood. This study aimed to uncover the molecular drivers behind solar lentigo progression, leveraging a novel network analysis approach to explore the dynamic molecular changes and key biomarkers involved in the disease.

Network analysis has been widely used alongside the emergence of big data, including transcriptomics, proteomics, and metabolomics. Traditional network analysis techniques mainly focus on metrics such as node degree, betweenness, and centrality to identify key nodes within biological networks. In contrast, the DNB methodology was specifically designed to detect the tipping point before the state transition based on “differential fluctuations” rather than traditional “differential expressions” for molecules, thus identifying warning signals by distinguishing dynamic network changes from static networks. DNB infers network fluctuations over time, providing valuable insights into biological systems and signaling potential system transitions. This approach has been successfully applied in a variety of contexts, including disease studies, cell fate decisions, immune checkpoint blockades, and aging. Importantly, DNB is not limited to time-series data but can also be used in multi-stage models.

In this study, we applied the DNB framework to a three-stage model of solar lentigines—photo-protected, peri-lesional, and lesional skin—by treating these spatially distinct but pathologically progressive stages as a pseudo-temporal sequence. Although this discrete approximation differs from traditional time-series data, it is biologically reasonable and enables the inference of gradual network changes associated with disease progression. The DNB scores showed a monotonic increase across the stages, consistent with clinical and molecular worsening. We acknowledge the limitations of discrete staging and suggest that future longitudinal studies with a finer temporal resolution would better leverage the strengths of the DNB framework for early-warning-signal detection.

To improve our understanding of molecular interactions, we incorporated a directed network into the DNB framework, transforming it into DND. The directed network allows us to model the relationships between adjacent nodes, offering deeper insights into complex biological systems by capturing both direct and indirect regulatory influences between mRNAs and miRNAs. This addition strengthens the DNB method, enabling us to explore not only the molecular state at a given time but also the dynamic transitions that drive disease progression.

Our findings show that integrating network information with feature expression effectively differentiated photo-protected sites from solar lentigines, and further distinguished lesions from peri-lesion sites. The expression profiling data clearly differentiated photo-protected skin from solar lentigo lesions, with similar expression patterns observed between lesion and peri-lesion sites. However, the DND approach was able to further distinguish these stages by identifying a gradual increase in DND scores from control to lesion. This progressive shift aligns with the phenotypic changes observed in solar lentigines, confirming that DND captures the dynamic molecular changes underlying the disease. Among the DND candidates, several genes were reported to be changed in solar lentigines, such as *COL4A1*. COL4 was significantly decreased in solar lentigines as reported by Miyachi et al. [38]. These results highlighted the ability of DND to identify key molecular events that might be overlooked in traditional static analyses.

A total of 387 features, including 70 miRNAs, were identified as DND candidates, many of which are linked to skin functions such as barrier formation and cell migration. GO enrichment analysis revealed their involvement in developmental and differentiation processes, while IPA highlighted significant pathways such as Wnt/β-catenin, RAR, and others. Additionally, the increasing network density and higher degree distribution observed in lesion-stage networks emphasized the central role of DNBs in disease progression.

Through DND prioritization, we identified 45 candidate features, among which *ARNT2* and *TBX2* emerged as key genes selected for in vitro validation. While *ARNT2* is not classically recognized as a core pigmentation regulator, prior studies suggest it may play a broader role in cell fate and proliferation. For example, in glioblastoma, *ARNT2* has been shown to be highly expressed in proliferative subpopulations and to promote tumorigenicity through the regulation of key transcription factors such as *SOX9*, *POU3F2* (*BRN2*), and *OLIG2* [39]. Given the shared neuroectodermal origin of glioblastoma and melanocytes, one study raises the possibility that *ARNT2* may influence melanocyte biology and pigmentation through similar developmental or stress-responsive pathways [32]. In our study, *ARNT2* showed consistently elevated expression across the stages of solar lentigines and a high network out-degree, supporting its potential relevance in pigmentation dynamics. *TBX2*, a member of the T-box family of transcription factors, has stronger support in the literature in both melanogenesis and melanoma biology. *TBX2* (and its paralog *TBX3*) are known to suppress cellular senescence through the repression of key cell-cycle regulators such as *ARF* and *p21* (*CDKN1A*) [33]. In melanoma cells, *TBX2* recruits *HDAC1* to the *p21* promoter, silencing its expression and enabling continued proliferation—even in the absence of *CDKN2A*, a well-known tumor suppressor frequently mutated in melanoma [33,34]. Additionally, *TBX2* and *TBX3* have been shown to bind both E-box and T-box motifs to regulate melanogenesis-related genes and bypass senescence, suggesting a dual role in pigmentation and oncogenesis [40]. The overexpression of *TBX2* in melanoma and other cancers such as breast, pancreatic, and liver cancer further supports its function as a key transcriptional repressor involved in maintaining a proliferative, pigmentation-prone state [33]. In summary, our findings propose *ARNT2* as a novel pigmentation-associated factor possibly linked to stress-response signaling, and highlight *TBX2* as a known transcriptional repressor with mechanistic links to melanocyte proliferation, senescence suppression, and pigmentation regulation. These insights deepen our understanding of the molecular networks underlying solar lentigines and open avenues for targeted interventions.

Future studies should aim to validate these DNDs in larger and more diverse cohorts to enhance the generalizability of our findings. Investigating the functional roles of these biomarkers will provide a deeper understanding of the molecular mechanisms driving solar lentigines. Moreover, integrating other omics data, such as proteomics and metabolomics, into the DND framework could further refine our understanding of the regulatory networks involved in the disease, potentially paving the way for personalized medicine strategies.

It is important to note that, although a directed network is included, our findings are based on correlation analyses, which do not imply causality. Future research using causal inference methods, such as Mendelian randomization or perturbation experiments, is necessary to determine whether these DNDs directly contribute to the observed phenotypic changes. Establishing causal relationships will be crucial for the development of targeted therapies based on these biomarkers. A potential limitation of our approach lies in the use of the mFVS algorithm, which may favor highly connected hub genes. While this could bias the results against less-connected but biologically relevant regulators, the purpose of mFVS is to identify a minimal set of nodes whose removal disrupts all feedback loops, thereby exerting maximum control over the network dynamics. Therefore, the presence of hubs among the prioritized genes may reflect their central regulatory roles rather than a purely topological bias. Nonetheless, we recognize that important low-degree nodes may be underrepresented. Future efforts could integrate biological priors or complementary ranking strategies to provide a more balanced and comprehensive identification of key regulators.

In conclusion, our study presents an innovative and integrative approach to understanding the molecular progression of solar lentigines. The DND method, combining classical DNB with directed network analysis, offers a powerful tool for identifying key biomarkers and potential therapeutic targets. This approach provides a solid foundation for future research and clinical applications, with the potential to improve the management and treatment of solar lentigines.

## 5. Conclusions

In this study, we presented Dynamic Network Driver (DND), a novel systems biology approach that integrates Dynamic Network Biomarker analysis with directed network control theory to identify key regulatory drivers of solar lentigo progression. By applying DND to multi-omics data across different stages of solar lentigines, we captured the temporal and network-level changes underlying disease progression. Our results revealed a progressive increase in DND scores across stages, consistent with phenotypic worsening, and identified *ARNT2* and *TBX2* as master regulators validated in both melanocyte cultures and 3D skin models. These findings suggest that *ARNT2* may play a previously unrecognized role in pigmentation dynamics via stress-response pathways, while TBX2 functions as a transcriptional repressor linked to melanocyte proliferation and senescence suppression. The DND approach successfully differentiated subtle molecular changes beyond traditional expression profiling, offering a dynamic perspective on disease evolution. Overall, this work provides new insights into the regulatory architecture of solar lentigines and highlights DND as a powerful framework for discovering biomarkers and therapeutic targets in progressive skin disorders.

## Data Availability

The data from this study are available from the corresponding author upon reasonable request with the permission of Unilever.

## Supplementary Materials

The following supporting information can be downloaded at: https://www.mdpi.com/article/10.3390/biology14070876/s1, Supplementary Figure S1. Outlier detection based on PCA and Mahalanobis distance; Supplementary Table S1. DND candidates list; Supplementary Table S2. DNDs list.
