# Supplementary material for: Dynamic Network Driver Analysis Identifies Master Factors Associated with Progression of Solar Lentigines
Source: Biology (Basel). 2025 Jul 17;14(7):876. doi: 10.3390/biology14070876 (PMC12292655; doi:10.3390/biology14070876)
Supplement: Supplementary file 1 [file biology-14-00876-s001.zip › biology-3732477 Supplementary Figure.pdf]

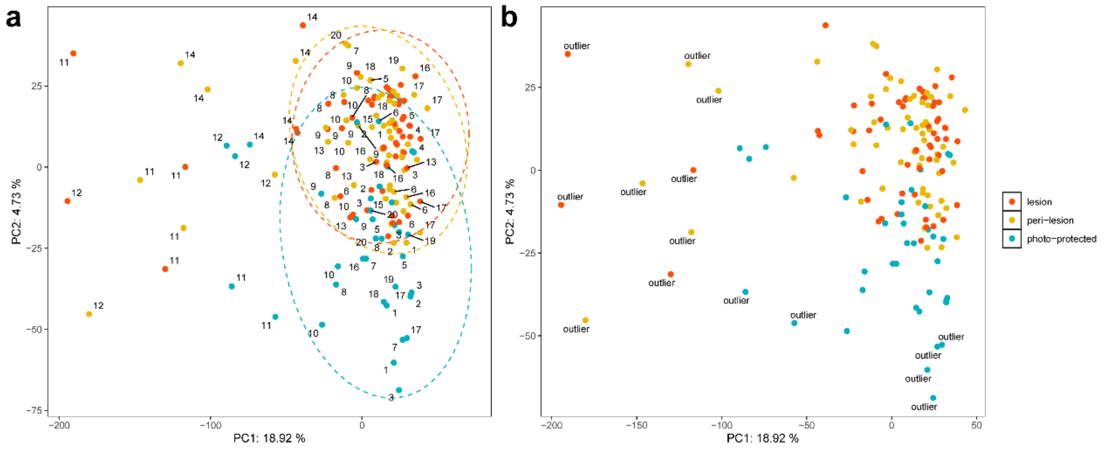

Supplementary Figure S1. Outlier detection based on PCA and Mahalanobis distance. (**a**) PCA plot of the combined mRNA and miRNA expression matrix for all 156 samples. Each point represents a sample, color-coded by stage (lesion, peri-lesion, and photo-protected). Ellipses indicate the 95% confidence region for each stage. A subset of samples from subjects 11, 12, and 14 that fall outside the ellipses were flagged as potential outliers. (**b**) Mahalanobis distance-based outlier detection in PCA space. Samples exceeding the 95% confidence threshold are marked as outliers. A subset of samples from subjects 1, 3, 7, 11, 12, 14 and 17 were flagged as potential outliers.
